# Detecting SARS-CoV-2 Variants in Wastewater and Their Correlation With Circulating Variants in the Communities

**DOI:** 10.21203/rs.3.rs-1435729/v1

**Published:** 2022-03-17

**Authors:** Lin Li, Timsy Uppal, Paul D. Hartley, Andrew Gorzalski, Mark Pandori, Michael A. Picker, Subhash C. Verma, Krishna Pagilla

**Affiliations:** University of Nevada; University of Nevada, Reno School of Medicine; University of Nevada; Nevada State Public Health Laboratory; Nevada State Public Health Laboratory; Southern Nevada Public Health Laboratory of the Southern Nevada Health District; University of Nevada, Reno School of Medicine; University of Nevada

**Keywords:** SARS-CoV-2, Wastwater-based epidemiology, Whole-genome sequencing, Variants, COVID-19

## Abstract

Detection of SARS-CoV-2 viral load in wastewater has been highly informative in estimating the approximate number of infected individuals in the surrounding communities. Recent developments in wastewater monitoring to determine community prevalence of COVID-19 further extends into identifying SARS-CoV-2 variants, including those being monitored for having enhanced transmissibility. We sequenced genomic RNA derived from wastewater to determine the variants of coronaviruses circulating in the communities. Wastewater samples were collected from Truckee Meadows Water Reclamation Facility (TMWRF) from November 2021 to June 2021 were analyzed for SARS-CoV-2 variants and were compared with the variants detected in the clinical specimens (nasal/nasopharyngeal swabs) of infected individuals during the same period. The comparison was found to be conclusively in agreement. Therefore, wastewater monitoring for SARS-CoV-2 variants in the community is a feasible strategy both as a complementary tool to clinical specimen testing and in the latter’s absence.

## Introduction

The pandemic caused by severe acute respiratory syndrome coronavirus 2 (SARS-CoV-2) has rapidly spread worldwide. In the early stage of the pandemic, Center for Disease Control and Prevention in the United States (US CDC) has called for a national collaboration of SARS-CoV-2 sequencing for public health emergency response, epidemiology, and surveillance (SPHERES), which can provide critical information about virus spreading, transmitting and evolution. This consortium includes over 160 universities, non-governmental organizations, and public health agencies. Sequencing all clinical specimens can be challenging due to the cost and the infrastructure to sequence many daily cases during a pandemic. Sequencing individual specimen samples were tedious and expensive. Therefore, alternative or complementary approaches may prove benefits in detecting circulating pathogens in the communities.

Molecular approaches are essential tools to determine the SARS-CoV-2 viral burden in a community by quantitation of viral RNA present in the wastewater, excreted from symptomatic and asymptomatic individuals ^2–5^. Wastewater is a pooled sample from the community at the sewershed scale and represents an overall prevalence of the SARS-CoV-2 in the same neighborhood ^6^. As a result, wastewater-based epidemiology (WBE) has been used as a complementary tool of SARS-CoV-2 environmental surveillance. It has shown great potential in identifying trends of COVID-19 numbers during the global pandemic ^7^. SARS-CoV-2 viral concentration in wastewater correlates positively with the clinically diagnosed number of COVID-19 cases ^4^. Therefore, it is reasonable to assume that sequencing of total environmental RNA from wastewater to detect the variants of SARS-CoV-2 is feasible and can help identify emerging variants. Additionally, this could increase the amount of genomic intelligence associated with SARS-CoV-2 variants that circulate in the community as a pool rather than individual specimens.

Efforts have been placed in identifying SARS-CoV-2 variants in wastewater, and a large number of variants have been detected and differentiated, including the variants of concern (VOCs) ^8–11^. VOCs are correlated with increased transmission and reduced effectiveness of the vaccines ^10,12,13^. Many studies have reported variants in wastewater. For example, B.1.1.7 variants have been identified in Israel since August 2020 ^14^. B.1.1.7, P.1, B.1.351, and B.1.617.2 have been identified in wastewater in March 2021, according to a study across 20 countries in Europe ^8^. These studies were conducted during the earlier wave of COVID-19 pandemic (September 2020 to February 2021 in Washoe County, Nevada, USA). One recent study from India detected Delta variant, which was considered the main reason for the second wave of this pandemic, through the analysis of SARS-CoV-2 signatures in the wastewater ^10^.

Herein, we describe the use of SARS-CoV-2 target capture strategies to enrich SARS-CoV-2 present in the wastewater for sequencing/variant determination and comparison with the variants detected by sequencing of SARS-CoV-2 in the clinical specimens. We combined the information gained from the sequencing of SARS-CoV-2 from individual-level variants identification with SARS-CoV-2 signatures obtained from the sequencing of wastewater to obtain the correlates for WBE. For the WBE, genomic material from the wastewater of the Reno-Sparks metropolitan, Nevada, USA, from November 2020 to June 2021 was analyzed to determine the viral load and variant determination through sequencing the whole SARS-CoV-2 genome. Our data shows that wastewater-based sequencing can be used to generate genomic epidemiologic intelligence that is equivalent to that gained through sequencing of individual clinical specimens.

## Methods And Materials

### Study area and SARS-CoV-2 quantification in wastewater

**Figure 1:**
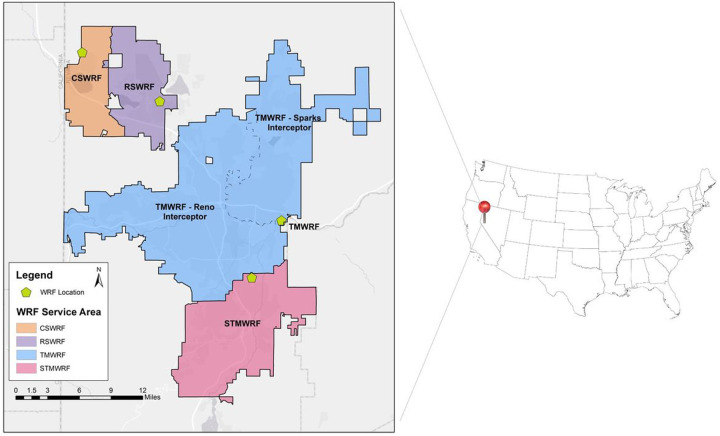
WRF service areas in Washoe County, NV, USA. Sampling occurred at the influent sampling head station of Truckee Meadows WRF (TMWRF), South Truckee Meadows WRF (STMWRF), Reno-Stead WRF (RSWRF), and Cold Spring WRF (CSWRF).

The study area of this research was the Reno-Sparks metropolitan area in Nevada (NV), USA, with a service population of 360,000. Influent wastewater from three water reclamation facilities (WRFs) was collected and analyzed since November 2020. Truckee Meadow Water Reclamation Facility (TMWRF) is the largest WRF in this area, with approximately 121,000 m^3^/day flowrate. TMWRF receives wastewater through two interceptor sewer lines, one routed from the Sparks metropolitan area, serving a population of 116,000 (indicated as TMWRF-North interceptor). A second sewer interceptor line is routed from the Reno metropolitan area, serving a population of 204,000 (shown as TMWRF-South interceptor). Mixed flow of both interceptors then directed to the main headworks, where wastewater flow has been sustained at approximately 96,000 m^3^/day during the sampling study period. South Truckee Meadow Water Reclamation Facility (STMWRF) served about 52,000 people in south Reno, Nevada. Reno-Stead Water Reclamation Facility (RSWRF) is the smallest WRF of the three sampling sites. As the facility serves a population of 18,000, it manages approximately 4% of the total wastewater generated in the sampling region. Figure 1 shows the sewershed of the sampled WRFs.

1L untreated grab wastewater samples after preliminary treatment were collected from three facilities. Grab samples were collected late morning between 9:00 am to 12:00 noon and transported directly to the laboratory on ice. Samples were kept at 4°C until further treatment for analysis. Samples were centrifuged at 3,000 × g for 15 min, and the resulting supernatants were sequentially filtered through 1.5, 0.8, and 0.45 μm sterile membrane filters to remove debris and large particles. The resulting liquid was used to concentrate the viruses. The virus concentration method was ultrafiltration using 100 KDa Amicon® Ultra-15 Centrifugal Filter Cartridge Units (Millipore Sigma, St. Louis, MO, USA). 30 mL samples were processed depending on the concentration level of viruses inside the wastewater. The purpose is to concentrate the viruses to a detectable level. After ultrafiltration, ~ 500 μL of concentrate was collected in each cartridge. The viral concentrates were typically stored at −80°C until downstream analysis unless analyzed the same day.

According to the user’s manual, total RNA from the concentrated samples was extracted from the AllPrep PowerViral DNA/RNA kit (QIAGEN, Inc., Germantown, MD, USA). Reverse transcription and quantitative polymerase chain reaction (RT-qPCR) was performed on the CFX96 Touch Real-Time PCR Detection System (BioRad, Hercules, CA, USA). Briefly, each reaction contained 5 μL of the 4× Reliance One-Step Multiplex Supermix (BioRad, Hercules, CA, USA), 5 μL of the total genomic RNA template, probes (0.2 μM) and primers (0.4 μM each) in a total volume of 20 μL. RT-qPCR was carried out according to the following program: reverse transcription at 50°C for 10 min, denaturation at 95°C for 10 min, followed by 45 cycles of 3 seconds denaturation at 95°C, 30 seconds annealing/extension and plate read at 60°C. Threshold cycle (Ct) was determined using the default algorithm in CFX Manager Software (BioRad, Hercules, CA, USA). The RT-qPCR assay used N1 and N2 primers and probes as per US CDC recommendation ^15^. Positive and non-template controls were included in each run. The field and RNA extraction blank were included monthly. Calibration curves (0 to 5-log range) were generated with 10-fold serial dilutions of SARS-CoV-2 positive control (IDT, Coralville, IA, USA) in the range from 200,000 to 2 gc/μL. Correlation coefficients (R2) > 0.99 were obtained for all calibration curves, with 90–110% amplification efficiencies. The limit of detection (LoD) was > 2 gc/μL of RNA elute in each qPCR assay, showing more than 50% positive signals with available Ct values of the lowest dilution of the positive control.

### COVID-19 clinical specimen collection

Nasal (N) Nasopharyngeal (NP) swabs of the deidentified human specimens were used to sequence the SARS-CoV-2 genome to determine the variants circulating in the communities in Reno/Sparks, NV, USA. The University of Nevada, Reno Institutional Review Board (IRB) reviewed this project and determined this study to be EXEMPT from the IRB review according to federal regulations and University policy. The NP swabs received at the NSPHL from the Reno-Sparks metropolitan for SARS-CoV-2 diagnostic testing were subjected for RNA extraction using Virus Total NA Isolation Fast Kit (Apostle MiniGenomics, San Jose, CA, USA), followed by RT-PCR for the detection of SARS-CoV-2 genome using US Federal Drug Administration Emergency Use Authorization (FDA-EUA) diagnostic kits, as described previously ^16^.

### Library prepartion and sequencing

SARS-CoV-2 positive clinical specimens from the Washoe county, collected during the study period, were subjected to the Whole SARS-CoV-2 Genome Sequencing (WGS) on Oxford Nanopore Technology (ONT) using Clear Labs DX (ClearLabs, Inc., Carlos, CA, USA) platform, described previously ^17^. Briefly, genomic RNA from the specimens were reverse transcribed to cDNA followed by PCR amplification of specific regions (target capture) of the SARS-CoV-2 cDNA. A modified version of the ARTIC v3 primer panel (98 tiled primers in two pools) was used to capture target regions of roughly 400 bp through PCR reactions, as described previously ^18^. Before the SPRI bead cleanup, the resulting amplicons were pooled together to remove unused PCR components and amplicons outside the expected size range. The purified amplicons of each sample were barcoded and pooled together for library preparation with Oxford Nanopore specific adapters. An additional SPRI clean-up was performed to remove any unattached adapters from the sequencing libraries before loading them onto the Flow Cells along with sequencing reagents. The sequencing Read base calling was processed live on the GridION instrument. Data were uploaded to the Clear Labs Cloud for bioinformatic analysis, including reading quality/length filtering and consensus-based assembly. The resulting FASTQ and FASTA files were downloaded through Clear Labs WGS Web App (ClearLabs, Inc., Carlos, CA, USA).

For the identification of variants in the wastewater, RNA extracted from the wastewater samples with Ct values (35–37) in RT-PCR were treated with DNase I (QIAGEN, Inc., Germantown, MD, USA) for 30 minutes at room temperature before concentrating through RNeasy Minelute spin columns (QIAGEN, Inc., Germantown, MD, USA). These concentrated samples were converted into Illumina-compatible sequencing libraries using a QIAseq FX Single Cell RNA Library kit (QIAGEN, Inc., Germantown, MD, USA), as described previously ^16^. Subsequent amplification of other RNA (ribosomal) in these samples was reduced by annealing with QIAseq FastSelect -HMR probes (QIAGEN, Inc., Germantown, MD, USA). RNA was reverse transcribed to cDNA using random hexamers, and then synthesized cDNA was ligated to one another, followed by isothermal linear amplification. Amplified DNA was enzymatically sheared to an average length of 300 bp, followed by ligating with Illumina-compatible dual-indexed sequencing adapters. These adapter-ligated samples were amplified with six cycles of PCR with KAPA HiFi HotStart polymerase (Roche Sequencing Solutions, Rotkreuz, Switzerland). SARS-CoV-2 specific sequences in these libraries were enriched with a myBaits kit and coronavirus-specific biotinylated probes (Arbor Biosciences, Ann Arbor, MI, USA) from approximately 500 ng of PCR-amplified DNA through hybridization at 65°C for 16 hours. DNA was further amplified by 8–16 cycles of PCR using KAPA HiFi HotStart polymerase. Samples were sequenced using an Illumina NextSeq mid-output (2 × 75) or NextSeq 2000 P2 100 cycle (2×50). The generated FASTQ files from the sequencing reaction were subjected to the detection of variant signatures.

According to the manufacturer’s directions, RNA from the wastewater sample collected in March 2021 was processed using the Illumina RNA Prep with Enrichment protocol (Illumina, Inc.). Briefly, total RNA was reverse transcribed to cDNA, tagmented, and then amplified. Next, the resulting amplified and tagmented cDNA was normalized, consolidated into singleplex or 3-plex samples, and then processed. Respiratory Virus Oligo Panel (RVOP) Enrichment Oligos labeled with biotin were used to enrich SARS-CoV-2 and other respiratory viral pathogens, captured with streptavidin-coated beads, washed. Capture cDNA was amplified and cleaned up. The resulting enrichment pools were quantified and diluted to a final concentration of 11.5 pM and run on an Illumina MiSeq instrument 2 × 75 cycles. FASTQ files from the sequencing reaction were used for variant identification.

### Bioinformatics pipeline for data analysis

FASTQ files generated from the Clear Dx (ClearLabs Inc., Carlos, CA, USA) were analyzed through cloud computing implemented in Terra with pipelines for lineage determination (pangolin), which is updated every 24 to 48 h to accommodate updated lineage defining mutations (Gorzalski et al., 2021). FASTQ files generated from the Illumina sequencing were analyzed using SARS-CoV-2 mutations analysis tool of the QIAGEN CLC Genomics Workbench (QIAGEN, Inc., Germantown, MD, USA). Workflow used to detect variants signature in wastewater samples is shown in Figure S1. Briefly, the sequence pair libraries were trimmed and mapped to SARS-CoV-2 (Wuhan-Hu-1) reference genome MN908947.3. Variants were called using Fixed Ploidy Variant Detection with ploidy = 1. We used a variant detection tool at minimum coverage and count to 10 and 5, respectively, with variant probably at 99%. Annotated variant tracks with amino acid changes were generated using the visualization tool. The number of reads for each allele was counted, and the percentages of the reads with single (SNV) or multiple (MNV) nucleotide variants were calculated based on the total reads for those alleles. Allelic signatures are present in the variants of both categories, the variant being monitored (VBM) or Variants of Concerns (VOC) of CDC (https://www.cdc.gov/coronavirus/2019-ncov/variants/variant-info.html), were determined in the sequences obtained from the wastewater.

## Results And Discussion

### SARS-CoV-2 monitoring in sampling locations

**Figure 2:**
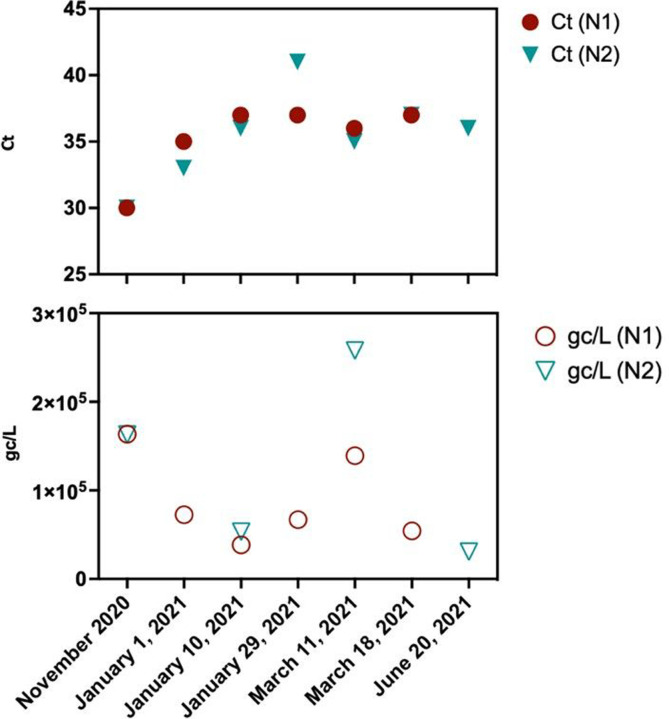
Detection of SARS-CoV-2 in the wastewater from November 2020 till June 2021, Ct values (top) with N1 and N2 primers in RT-qPCR, and the calculated genome copies/L (bottom) of of the wastewater. Samples collected on November 2020, January 1, 2021, March 11, 2021, and June 20, 2021 were used to detect SARS-CoV-2 variants through enrichment and sequence analysis.

SARS-CoV-2 monitoring through wastewater has been very informative and correlative to the number of COVID-19 cases. The SARS-CoV-2 wastewater surveillance in Washoe County, NV, was initiated early in the pandemic when the number of COVID-19 cases was starting to increase. Consequently, rising levels of SARS-CoV2 (detected through N1 and N2 genes) were observed in all three facilities influent wastewater since late September 2020, which correlated with an increase in the number of clinical COVID-19 cases in Washoe County ^4,19^. A detailed method of SARS-CoV-2 recovery from the wastewater was validated using current virus concentration methods by using the recovery control, inhibition control, and endogenous wastewater control ^20^. More than 600 samples from three facilities were analyzed over 15 months, and concentrations of gene copies ranged between 3.00 × 10^2^ to 9.28 × 10^5^ gc/L. We detected an increase in the concentrations of SARS-CoV-2 in wastewater with Ct values as low as 30.31, corresponding to 1.6×10^5^ genome copied/L (Fig. 2 and Table S2). Therefore, we used wastewater samples from November 2020 onwards to detect SARS-CoV-2 signatures with an interval of 2 months (November 2020, January 2021, March 2021, and June 2021). SARS-CoV-2 variant signatures detected in wastewater were compared with the variants detected in COVID-19 clinical specimens during the same period.

### Detection of SARS-CoV-2 variants through wastewater based epidemiology

While the viral load in the wastewater positively correlates with the number of SARS-CoV-2 infected individuals, determining the lineage of circulating variants can further provide intelligence on whether any specific variants change over time in the communities. SARS-CoV-2 variants impacting approved or authorized medical countermeasures or associated with more severe disease or increased transmission are categorized as variants of interests. However, when such variants are no longer detected or circulate at very low levels, posing minimal risk to public health, they are categorized under the monitored variants (VBM). Here, we hypothesized that SARS-CoV-2 variant signatures in wastewater positively correlate with circulating variants in humans. We tested our hypothesis by analyzing SARS-CoV-2 sequences in the clinical samples collected during the same period as the wastewater. Our site for the correlative study of variants in the wastewater and humans in the Reno-Sparks metropolitan area provides a decently controlled environment with a minimal influx of wastewater contribution from visitors, which may interfere with the signatures at a given time.

**Figure 3:**
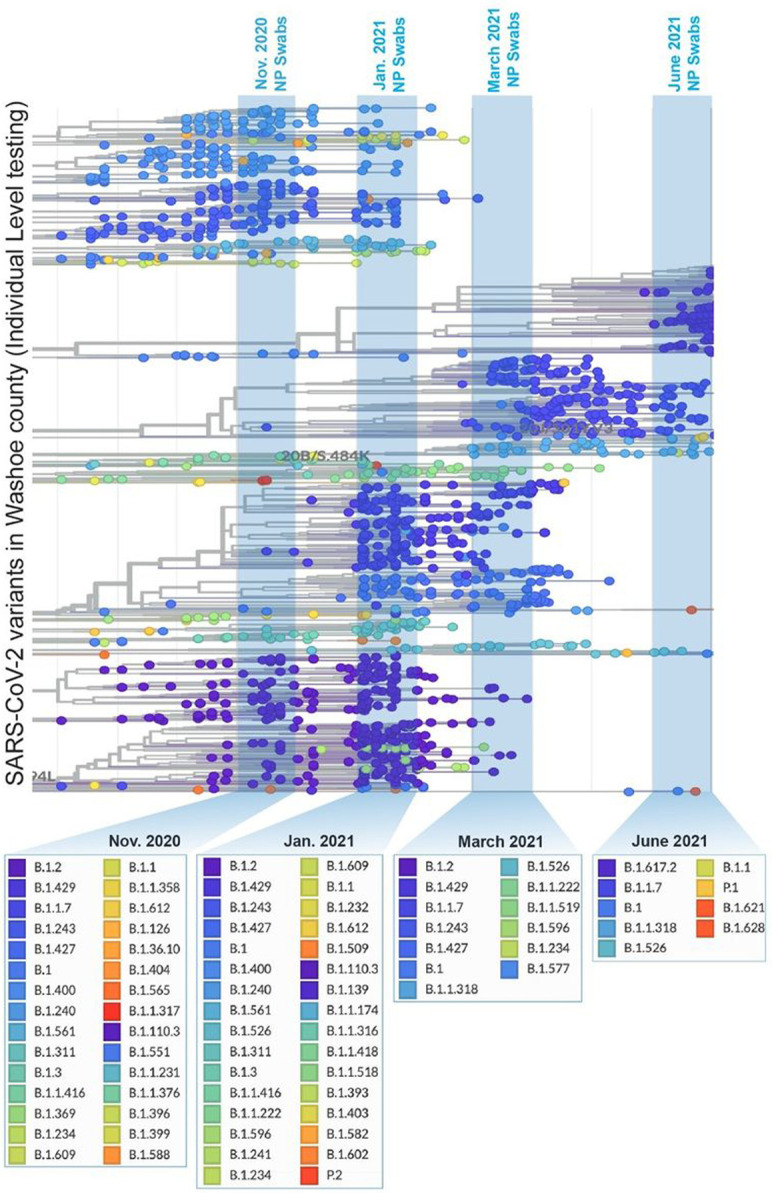
Circulated SARS-CoV-2 variants in the community (Washoe County, Nevada, USA), detected through sequencing of clinical specimens of COVID-19 patients during November 2020, January 2021, March 2021, and June 2021.

We began analyzing the wastewater sample of the most recent collection (June 20, 2021, for the presence of SARS-CoV-2 signatures, which identified many variants defining signatures of SARS-CoV-2 in the wastewater (Table S3). To determine the circulating variants, we generated a snapshot of SARS-CoV-2 variants present among individuals with SARS-CoV-2 infection by sequencing the clinical samples (N or NP swabs) of the Reno-Sparks metropolitan area at the NSPHL. SARS-CoV-2 sequences of the randomly selected clinical samples from the individuals of the Reno-Sparks metropolitan area during November 1, 2020, and June 30, 2021, were displayed through www.auspice.us (genomic epidemiology of novel coronavirus built on www.nextstrain/ncov) (Fig. 3). Lineages of SARS-CoV-2 circulated during the indicated months are highlighted on the phylogenetic tree (Fig. 3). The list of SARS-CoV-2 variants circulated during those months of wastewater genome surveillance are shown below the indicated months (Fig. 3). The diversity of variants decreased over time after introducing highly transmissible variants, B.1.1.7 and B.1.617.2. as expected due to the high transmission rate. Analysis of SARS-CoV-2 variants circulating in June 2021 was predominantly B.1.617.2 (Delta variant) with B.1.1.7 as the second predominant variant in the Reno-Sparks metropolitan area (Fig. 3). During March 2021, the most circulated variant was detected to be B.1.2, however lack of granularity for lower order lineage classification may have categorized them under lineage B.1.2. The other dominant variants, B.1.429 and B.1.1.7 during that time periods were classified under the category of VOIs or VOCs, respectively and these are currently under VBM. SARS-CoV-2 variants during January, 2021 and November, 2020, belonged to a large groups of variants (Fig. 3).

**Figure 4:**
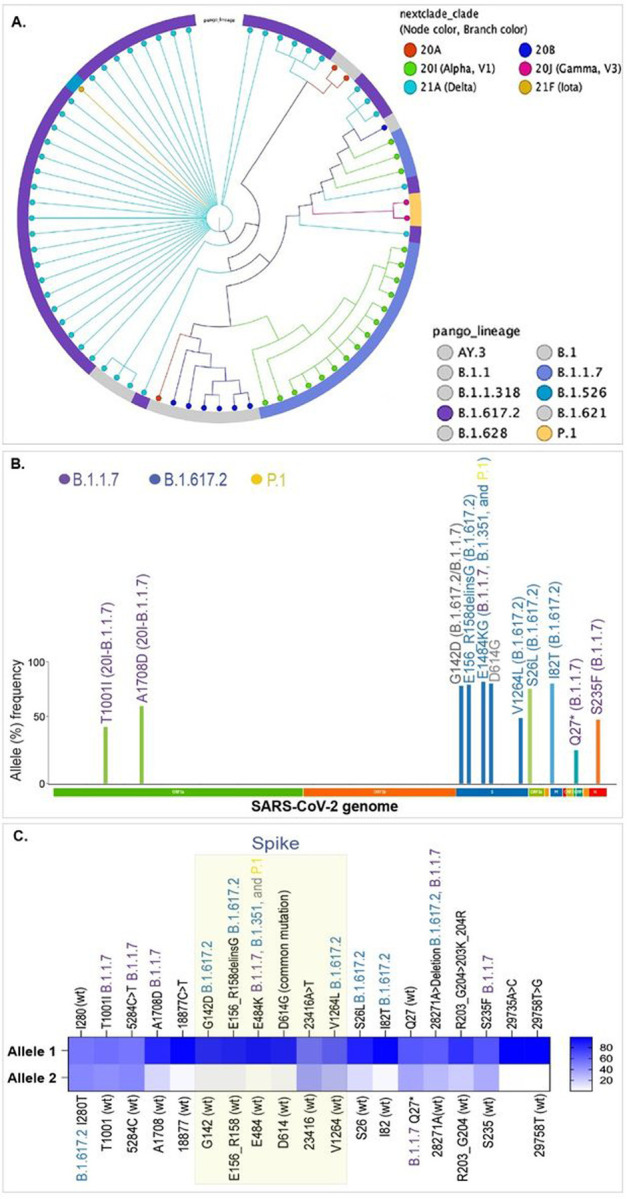
Detection of SARS-CoV-2 specific signatures in the wastewater and their correlation with the variants circulating in the community during June 2021. **A.** Lineage distribution of SARS-CoV-2 variants circulated in the community identified through individual level testing. These variants are classified based on the Nextclade as well as pangolin lineages. **B.** Relative allele frequencies of the key mutations, important in defining variants, detected in the wastewater during June 2021. **C.** Heat-map shows the variant’s relative abundance defining allelic mutations in the sequence reads. Allele 1 mutation was present in the most number of reads. Allele 2 was present in the remaining reads covering those indicated mutations.

With an aim to maximize the detection of variants circulating in Washoe county, we performed enrichment and sequencing of SARS-CoV-2 of the wastewater during the above time period and compared with the variants detected through individual level testing of clinical specimens from the community. Lineage classification of variants in clinical specimens collected during June, 2021 showed predominantly B.1.617.2 (21A) and B.1.1.7 (20I), presented as a K-mer phylogenetic tree with relative proportions of each detected variants (Fig. 4). The variant signatures detected in wastewater through the variants calling tool of the CLC Genomic Workbench by comparing with Wuhan-Hu-1 SARS-CoV-2 are displayed in the associated_variant_track panel (Figure S2A). A list of all detected variants along with the count and coverage of each allele is presented (Table S3). The number of reads (count) and genome coverages for each samplewere determined using the CLC Workbench. Allelic frequencies (%) depicting the relative abundances, calculated based on the count and coverages of the reads, detected variants defining signatures of B.1.617.2 (21A) and B.1.1.7 (20I) clades (Fig. 4B). Relative prevalences of variants signatures in the wastewater showed a higher level of B.1.617.2 (21A) defining signatures, which is congruent with the percentages of SARS-CoV-2 cases determined by the individual level testing of clinical samples (Fig. 4C). Importantly, we were able to detect the signatures of low abundant variant, P.1 through the Wastewater Based Epidemiology (Fig. 4). This conclusively showed that WBE can detect the signatures of even low abundantly present SARS-CoV-2 variants and thus can be useful for detecting VOIs/VOCs signatures early in their spread.

**Figure 5:**
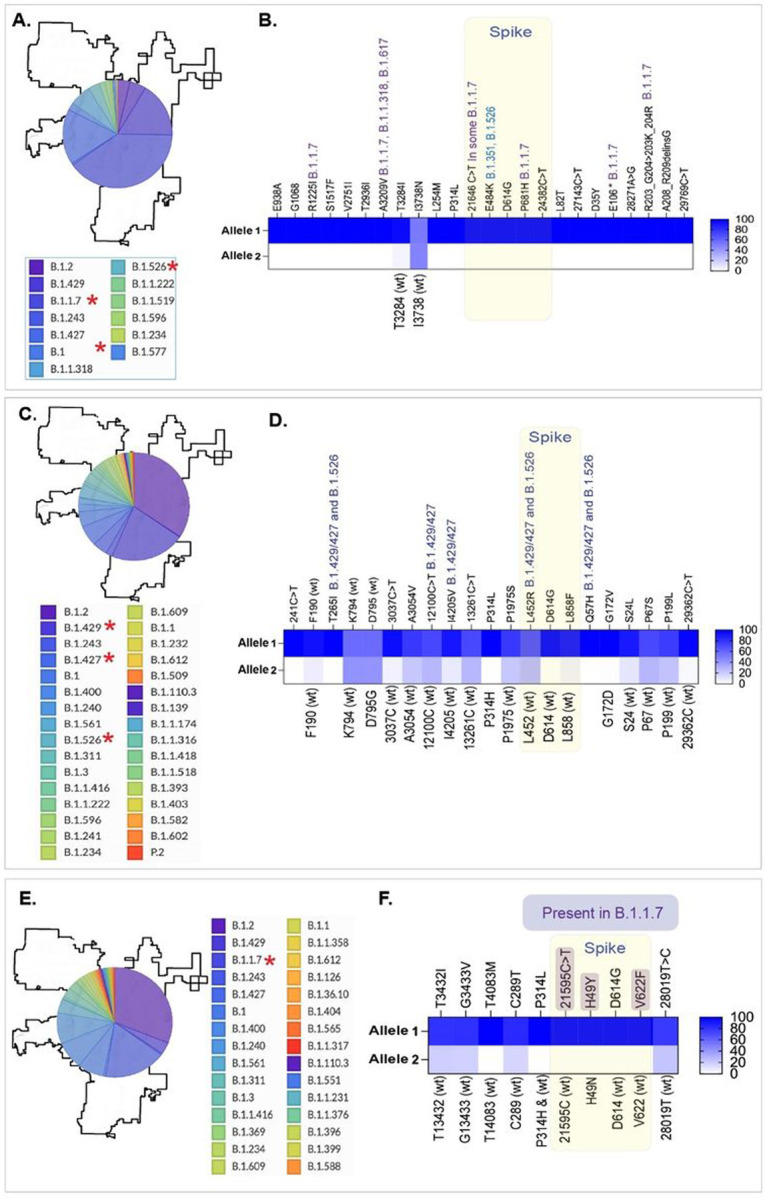
Detection of SARS-CoV-2 variants specific signatures in wastewater and their correlation with circulating variants identified through individual level testing during three months. **A.** Relative abundance of SARS-CoV-2 variants was detected through individual level testing during March 2021. **B.** Variant defining mutations of the SARS-CoV-2 genome detected in the wastewater during March 2021. **C.** Relative abundance of SARS-CoV-2 variants in the Washoe county detected through individual level testing during January 2021. **D.** Variant-defining mutations of the SARS-CoV-2 genome detected in the wastewater during January 2021. **E.** Relative abundance of SARS-CoV-2 variants in the Washoe county detected through individual level testing during November 2020. **F.** Variant defining mutations of the SARS-CoV-2 genome detected in the wastewater during November 2020.

Next, we determined whether the WBE functions is predictive of identifying variants over a period of time, by analyzing the diversity of SARS-CoV-2 variants in the wastewater collected at three additional time points, March, 2021, January, 2021 and November, 2020. The reads mappings and variant signatures detected in the wastewater collected during these months are shown as supporting data (Figures. S2B–D) along with the number of reads for each allelic variant including the variant-defining alleles (Tables S4–6). Relative abundances of SARS-CoV-2 variants detected through the individual level testing are presented as pie charts for those three indicated months (Figures. 5A, C and E). Allelic frequencies of variant-defining signatures in WBE were plotted as heat-maps, which showed the signatures of all the variants of interests as well as the variants of concerns (now variants being monitored) at three indicated time points (Figures. 5B, D and F). These variants are marked with red asterisks and the signatures of those VBM in the wastewater are marked above those mutations (Fig. 5). B.1.1.7 (20I) were among the predominant variants circulating during March, 2021 in Washoe County, which corelated with the highest number of reads for mutations defining B.1.1.7 (20I) variant (Figures. 5A and B). Similarly, B.1.427/429 (Epsilon) variants was highly prevalent in the month of January, 2021, also detected by the number of reads for the allelic mutations defining B.1.427/429 (Fig. 5C and D). We also detected the signatures of in B.1.1.7 through WBE in November, 2020, when the diversity of circulating mutations was high (Figures. 5E and 5F). This supports our hypothesis that WBE detects circulating SARS-CoV-2 variants in the community.

### Comparison with SARS-CoV-2 variants monitoring in wastewater and the variants from clinical sequencing results

The predominant variants in the Washoe county during the month of June, 2021 were B.1.617.2, B.1.1.7 with a small proportion of B.1.526 and P.1 (Fig. 4). B.1.1.7, P.1 and B.1.617.2 belonged to the VOCs and B.1.526 to the VOIs, which are now under the category of VBM because of their low or almost no transmission but still requiring monitoring for their potential to countermeasure the approved therapeutics. When analyzing the frequencies of VBM signatures in the sequences of wastewater specimens, we found that the prevalences of these VOC/Is were correlated to their occurrences among community individuals. Percent frequencies of the reads with variants defining signatures for Alpha (B.1.1.7) and Delta (B.1.617.2) ranged between 80–90%, which was in the same range as the community prevalence of these variants during June, 2021 (Fig. 4). Similarly, during March 2021, Alpha (B.1.1.7) and Epsilon (B.1.429/427) were the most prevalent variants in the community, which was also reflected by the percent frequencies of these VBM specific signatures (Table S4). Lineage analysis of variants in the community during January 2021 showed a high diversity of variants with three VOIs (B.1.429/427 and B.1.526), which was also detected in wastewater samples collected during the same period (Table S5). Notably, the wastewater samples collected during November 2020, when only a few cases of the Alpha (B.1.1.7) variants in the Washoe County, were detected to contain mutations associated with the Alpha variant (Table S6). Although these signatures were not Alpha variant-specific and the other Alpha variant specific mutations (Spike, N501Y) were in the region with low sequencing coverage. We suspect that low coverage was due to the quality of RNA stored for over six months, as we retrospectively analyzed samples from earlier time points for this comparative study.

## Conclusions

The data shown herein demonstrate wastewater-based methods’ efficacy in detecting and describing SARS-CoV-2 variants in an urban setting. Estimation of the presence of viral variants by wastewater-based methods reflected estimates generated from individual sequencing of clinical specimens. Sequencing individual clinical samples provide higher quality sequence data, but reliance on surveillance on such specimens alone has a weakness. Wastewater is less dependent on a populace’s willingness to test. Moreover, it allows the examination of communities where testing data may be limited. WBE may indicate a snapshot of all the SARS-CoV-2 variants circulating in the community during the time of sample collection. Routine analyses of wastewater could provide ongoing surveillance of existing lineages and may even detect novel lineages for a community.

## Supplementary Material

1

2

## Data Availability

Sequencing reads are submitted to Genbank with BioProject ID: PRJNA772783 (https://www.ncbi.nlm.nih.gov/bioproject/772783). SARS-CoV-2 sequences from the Individual level testing are available at GISAID, and their accession numbers are provided in Table S1.
