# Supplementary material for: Detecting SARS-CoV-2 Variants in Wastewater and Their Correlation With Circulating Variants in the Communities
Source: Res Sq. 2022 Mar 17:rs.3.rs-1435729. Preprint. [Version 1] doi: 10.21203/rs.3.rs-1435729/v1 (PMC8936115; doi:10.21203/rs.3.rs-1435729/v1)
Supplement: 1 [file 78d5f5680cc7a9cfff8089d3.docx]

**Supplementary Information**

**Detecting the prevalence of SARS-CoV-2 Variants in the wastewater and their correlation with circulating variants in the communities**

Lin Li^1^, Timsy Uppal^2^, Paul D. Hartley^3^, Andrew Gorzalski^4^, Mark Pandori^4,5^, Michael A. Picker^6^, Subhash C. Verma^2, *^, Krishna Pagilla^1, *^

*Affiliations:*

^1^ Department of Civil and Environmental Engineering, University of Nevada, MS258, Reno, NV 89557, USA

^2^ Department of Microbiology and Immunology, University of Nevada, Reno School of Medicine, MS320, Reno NV, 89557, USA

^3^ Nevada Genomics Center, University of Nevada, Reno, NV, 89557, USA

^4^ Nevada State Public Health Laboratory, Reno, NV, USA

^5^ Department of Pathology and Laboratory Medicine, University of Nevada, Reno School of Medicine, Reno, NV, USA

^6^Southern Nevada Public Health Laboratory of the Southern Nevada Health District, Las Vegas, NV, USA

^*^ To whom correspondence should be addressed:

**Subhash C. Verma** [**scverma@med.unr.edu**](mailto:scverma@med.unr.edu) and **Krishna Pagilla** [**pagilla@unr.edu**](mailto:pagilla@unr.edu)


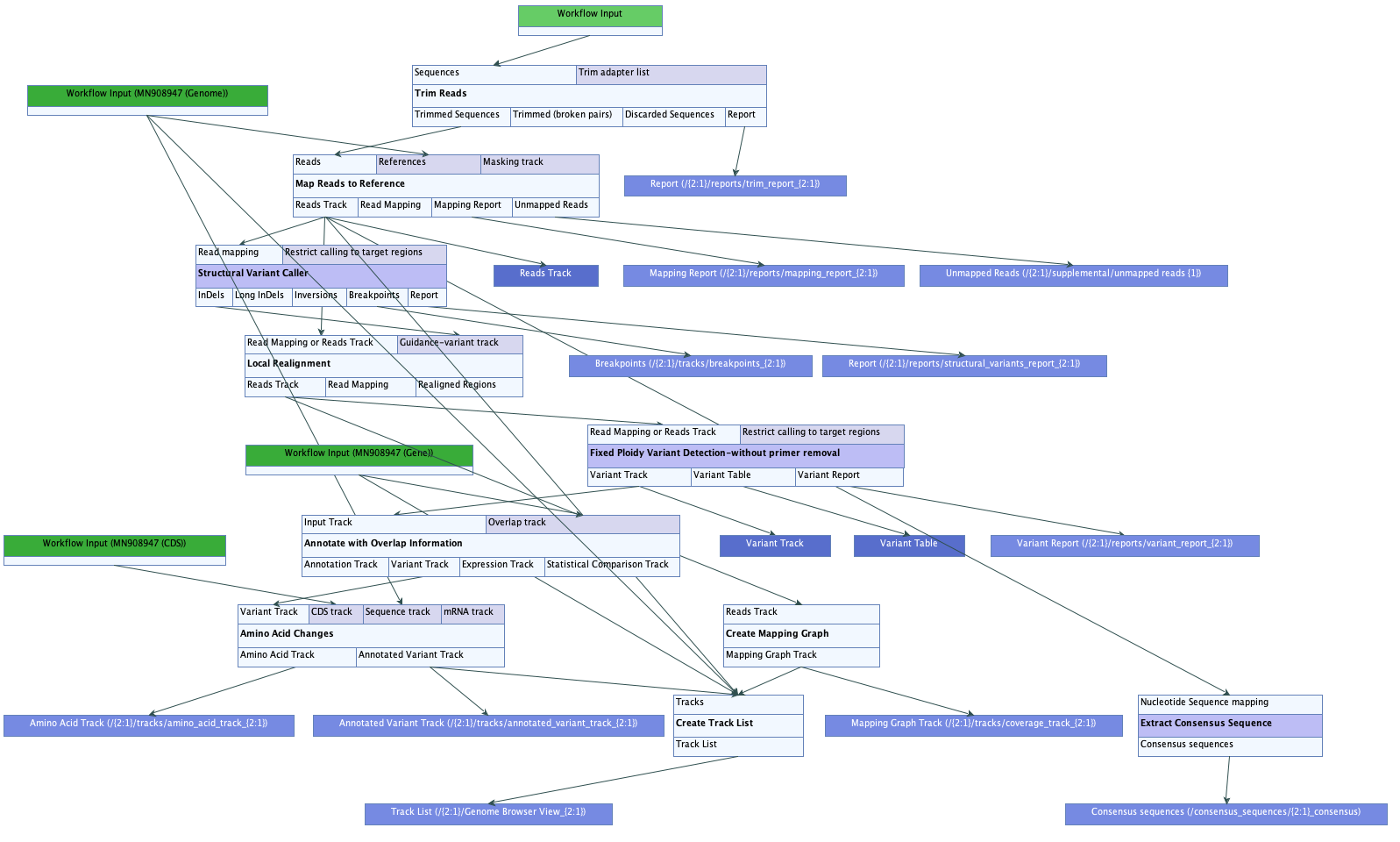


Supplementary Figure 1. Workflow used for the identification of variants of CLC Genomic Workbench v21.


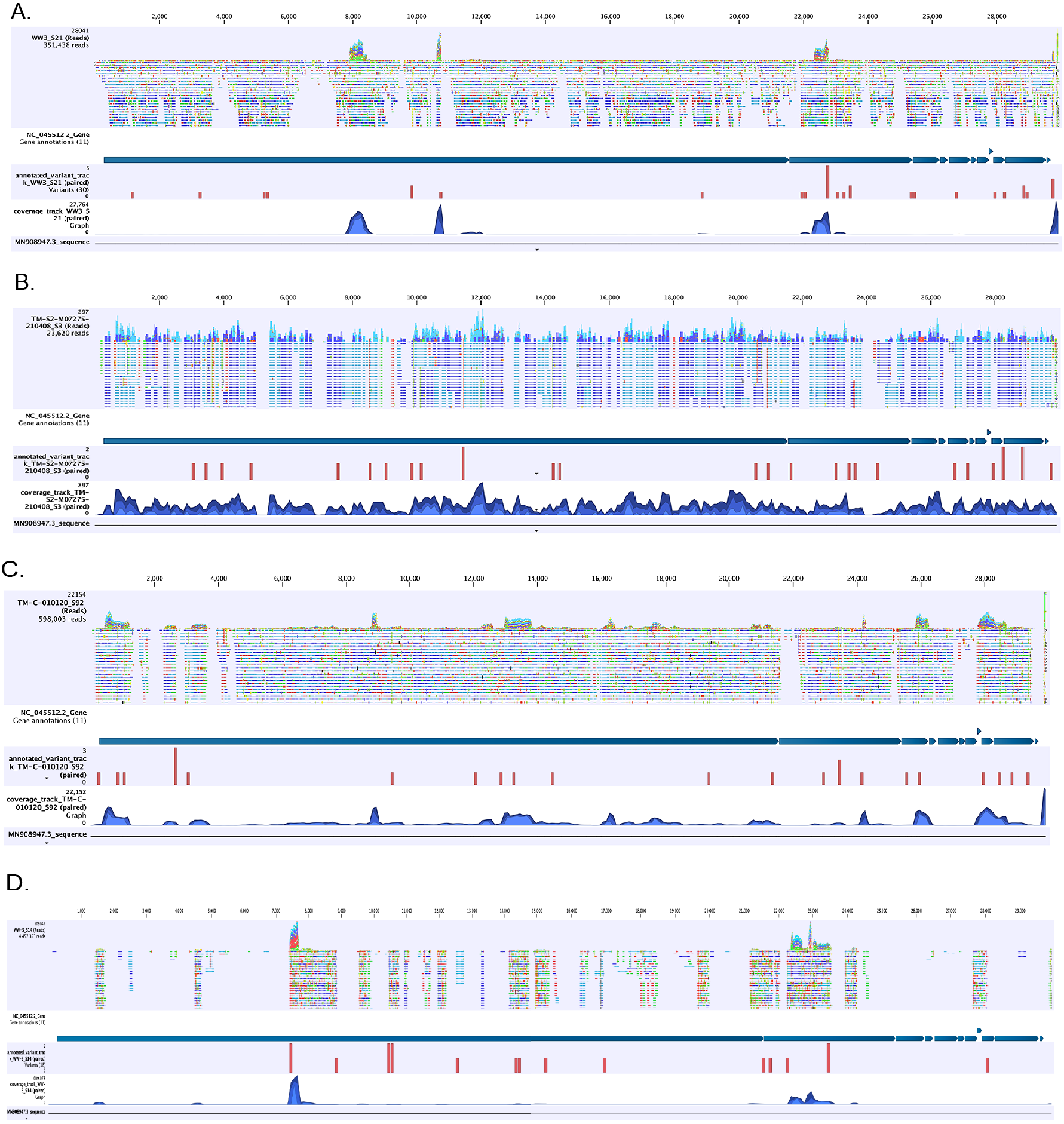


Supplementary Figure 2. Mapping of the reads from wastewater specimens to the reference SARS-CoV-2 (Wuhan-Hu1), accession number NC_045512.2. **A.** Sequence reads in the wastewater from June, 2021; **B.** Sequence reads in the wastewater from March 2021; C. Sequence reads in the wastewater from January 2021; D. Sequence reads in the wastewater from Novemver 2020.
